# Trends in Buprenorphine Use in US Jails and Prisons From 2016 to 2021

**DOI:** 10.1001/jamanetworkopen.2021.38807

**Published:** 2021-12-14

**Authors:** Ashish P. Thakrar, G. Caleb Alexander, Brendan Saloner

**Affiliations:** 1 National Clinician Scholars Program at the Corporal Michael J. Crescenz Veterans Affairs Medical Center, University of Pennsylvania, Philadelphia; 2 Leonard Davis Institute of Health Economics, University of Pennsylvania, Philadelphia; 3 Johns Hopkins Bloomberg School of Public Health, Baltimore, Maryland

## Abstract

This cross-sectional study examines trends in the use of buprenorphine among incarcerated individuals in the US.

## Introduction

An estimated 15% of the 1.8 million incarcerated individuals in the US have opioid use disorder (OUD).^1,2^ These individuals have a substantially higher risk of overdose after leaving correctional facilities.^1^ Pharmacotherapy for OUD is associated with reductions in postincarceration mortality, yet as of 2018, less than 14% of correctional systems offered buprenorphine or methadone.^3^ Over the past 5 years, more municipalities and states have enacted policies to provide access to OUD treatment, but the extent to which this implementation has actually increased buprenorphine use remains unclear.^3^

## Methods

This cross-sectional study did not qualify as human participant research according to Johns Hopkins Medicine’s policy on deidentified data; thus, the study was considered exempt from review by the institutional review board and the requirement to obtain informed consent. We followed the Strengthening the Reporting of Observational Studies in Epidemiology (STROBE) reporting guideline.

We used previously published prevalence data to estimate the number of incarcerated individuals with OUD.^1,2^ Using data obtained from the National Sales Perspectives (IQVIA), we quantified US buprenorphine use overall and within correctional settings from June 1, 2016, through May 31, 2021. The National Sales Perspectives projects national pharmaceutical sales based on direct measurements of more than 90% of all retail and nonretail sales from manufacturers and wholesalers to pharmacies, clinics, hospitals, long-term care facilities, federal and state prisons, county and city jails, and juvenile detention centers.^4^ The National Sales Perspectives reports buprenorphine data in extended units without regional, prescriber, or patient-specific information, with each extended unit corresponding to 1 film, tablet, or injection. We converted sublingual extended units to milligrams of buprenorphine and treated 16 mg as 1 day of treatment given that doses of at least 16 mg daily reduce illicit opioid use.^5^ We treated each injection of extended-release buprenorphine as 30 days of treatment, regardless of dose. We excluded buccal, patch, and intravenous buprenorphine formulations because they are not indicated for OUD. We reported the daily mean number of treated individuals over a rolling, 3-month period.

Methadone was not examined because it is commonly delivered to carceral settings after being sold to community-based opioid treatment programs, and the National Sales Perspectives does not record these deliveries. Data analyses were conducted using Excel, version 16.53 (Microsoft Corp).

## Results

Buprenorphine use in jails and prisons increased by 224-fold, from a daily mean of 44 individuals in June 2016 to 9841 individuals in May 2021 (Figure). Most of this increase occurred from 2020 to 2021. Nationwide, across all retail and nonretail settings, buprenorphine use increased by 53.9% from a daily mean of 466 781 individuals in January 2015 to 718 591 individuals in May 2021. By May 2021, correctional settings accounted for approximately 1.5% of all buprenorphine use nationwide. An estimated 3.6% of the 270 000 incarcerated individuals with OUD in the US received buprenorphine.

**Figure.**
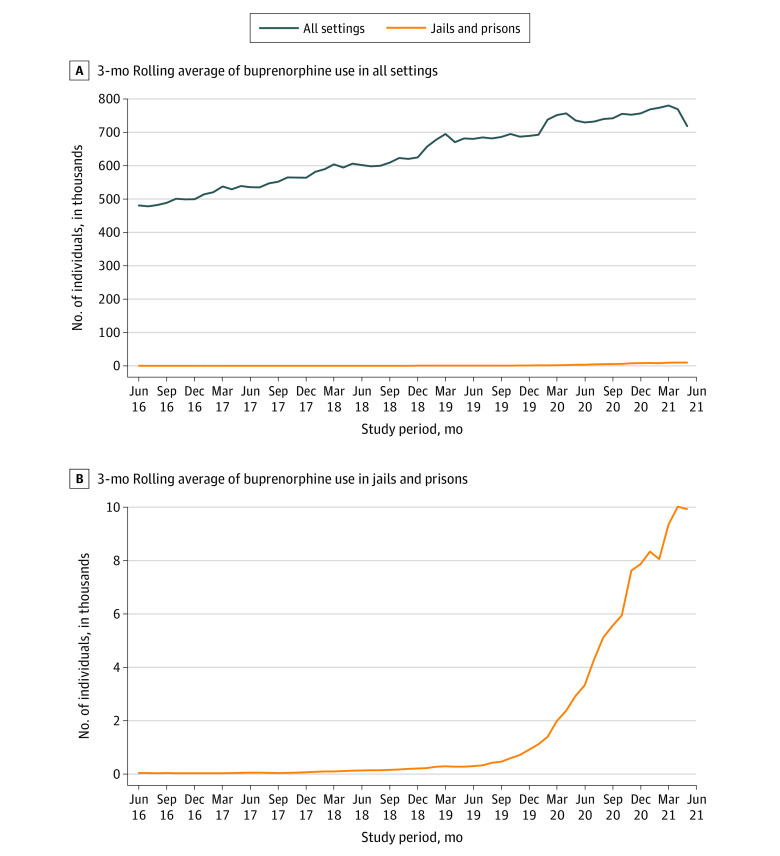
US Individuals Who Received Buprenorphine From 2016 to 2021

## Discussion

In the US, buprenorphine use increased substantially in correctional settings during the past 5 years, although use is still rare among all incarcerated individuals with OUD. Study limitations included the inability to account for supply chains that were excluded from the National Sales Perspectives data or carceral facilities that used community pharmacies to procure buprenorphine.

This increase in buprenorphine use represents progress, albeit incomplete, toward improving access to OUD treatment for incarcerated individuals. This progress was likely facilitated by federal-level guidance and support paired with state-level legislation and litigation regarding access to medications for OUD in correctional settings.^1^ Since 2019, the Substance Abuse and Mental Health Services Administration and the Bureau of Justice Assistance’s Comprehensive Opioid, Stimulant, and Substance Abuse Program have provided guidelines, resources, and technical assistance to expand the availability of pharmacotherapy for OUD in the criminal justice system.^3^ Meanwhile, a few states (eg, Rhode Island, Vermont, and Delaware) have recently mandated that jails and prisons offer medications for OUD.

Although this study found that buprenorphine use has increased, access in jails and prisons is still inadequate. Extensive efforts remain under way to expand the availability of all medications for OUD in carceral settings.^6^

## References

[1] Mancher M, Leshner AI, eds; National Academies of Sciences, Engineering, and Medicine; Health and Medicine Division; Board on Health Sciences Policy; Committee on Medication-Assisted Treatment for Opioid Use Disorder. Medications for Opioid Use Disorder Save Lives. National Academies Press; 2019. 30896911

[2] Kang-Brown J, Montagnet C, Heiss J. People in Jail and Prison in 2020. Vera Institute of Justice; 2021.

[3] Substance Abuse and Mental Health Services Administration. Use of Medication-Assisted Treatment for Opioid Use Disorder in Criminal Justice Settings. Substance Abuse and Mental Health Services Administration; 2019.

[4] IQVIA. Harness the power of real world data. Accessed October 7, 2021. https://www.iqvia.com/solutions/real-world-evidence/real-world-data-and-insights

[5] Mattick RP, Kimber J, Breen C, Davoli M. Buprenorphine maintenance versus placebo or methadone maintenance for opioid dependence. Cochrane Database Syst Rev. 2004;(3):CD002207. doi:10.1002/14651858.CD002207.pub4 15266465

[6] National Governors Association; American Correctional Association. Expanding Access to Medications for Opioid Use Disorder in Corrections and Community Settings: A Roadmap for States to Reduce Opioid Use Disorder for People in the Justice System. National Governors Association and American Correctional Association; 2021.

